# The effect of latent and error non-normality on corrections to the test statistic in structural equation modeling

**DOI:** 10.3758/s13428-021-01729-9

**Published:** 2022-01-10

**Authors:** Lisa J. Jobst, Max Auerswald, Morten Moshagen

**Affiliations:** Department of Psychological Research Methods, Institute of Psychology and Education, Ulm University, Albert-Einstein-Allee 47, 89081 Ulm, Germany

**Keywords:** Structural equation modeling, Non-normal multivariate data, Source of non-normality, Monte Carlo simulation, Corrections to the test statistic

## Abstract

In structural equation modeling, several corrections to the likelihood-ratio model test statistic have been developed to counter the effects of non-normal data. Previous robustness studies investigating the performance of these corrections typically induced non-normality in the indicator variables. However, non-normality in the indicators can originate from non-normal errors or non-normal latent factors. We conducted a Monte Carlo simulation to analyze the effect of non-normality in factors and errors on six different test statistics based on maximum likelihood estimation by evaluating the effect on empirical rejection rates and derived indices (RMSEA and CFI) for different degrees of non-normality and sample sizes. We considered the uncorrected likelihood-ratio model test statistic and the Satorra–Bentler scaled test statistic with Bartlett correction, as well as the mean and variance adjusted test statistic, a scale-shifted approach, a third moment-adjusted test statistic, and an approach drawing inferences from the relevant asymptotic chi-square mixture distribution. The results indicate that the values of the uncorrected test statistic—compared to values under normality—are associated with a severely inflated type I error rate when latent variables are non-normal, but virtually no differences occur when errors are non-normal. Although no general pattern regarding the source of non-normality for all analyzed measures of fit can be derived, the Satorra–Bentler scaled test statistic with Bartlett correction performed satisfactorily across conditions.

A crucial issue in structural equation modeling (SEM)—as in any statistical modeling technique—is the reliable evaluation of model fit to assess how well a particular model describes the data. In the context of SEM, the likelihood-ratio model test (LRT) statistic based on maximum likelihood (ML) estimation comparing the fit of the investigated model against the saturated model is the most widely used (Savalei & Kolenikov, 2008). The LRT statistic is derived based on the assumption that the observed variables follow a multivariate normal distribution. In case of non-normality, however, the type I error rates of the LRT statistic are—sometimes grossly—inflated (e.g., Curran et al., 1996; Maydeu-Olivares, 2017; Nevitt & Hancock, 2004). Given that the assumption of normally distributed data rarely holds in substantive research (e.g., Blanca et al., 2013; Cain et al., 2017; Micceri, 1989), several corrections to the LRT statistic have been developed aiming at modifying the test statistic to more closely follow the asymptotic chi-square distribution under conditions of non-normality (e.g., Asparouhov & Muthén, 2010; Lin & Bentler, 2012; Satorra & Bentler, 1994).

The performance of these corrections has been investigated in numerous robustness studies (e.g., Chou et al., 1991; Curran et al., 1996; Fouladi, 2000; Nevitt & Hancock, 2004; Maydeu-Olivares, 2017; Satorra & Bentler, 1994; Savalei, 2010; Tong & Bentler, 2013). Some of these studies suggest that the Satorra–Bentler scaled test statistic (Satorra & Bentler, 1994) in particular closely follows the underlying chi-square distribution. Nevertheless, tendencies to overreject a fitting model could be observed in small samples (e.g., Nevitt & Hancock, 2004; Maydeu-Olivares, 2017; Savalei, 2010; Tong & Bentler, 2013). However, applying the Bartlett (1950) correction to the Satorra–Bentler scaled test statistic seems to result in substantial improvements when only a few observations are available (e.g., Nevitt & Hancock, 2004; Savalei, 2010). Other simulation studies suggest that mean and variance-adjusted test statistics are a reasonable choice to deal with non-normal data in SEM: Whereas the mean and variance adjusted test statistic introduced by Asparouhov and Muthén (2010) seems to perform satisfactorily across a wide range of conditions (Maydeu-Olivares, 2017), the Satorra–Bentler adjusted test statistic (Satorra & Bentler, 1994) appears to be recommendable in small samples (Fouladi, 2000; Savalei, 2010).

Nevertheless, as the abovementioned studies focus on manipulating the distribution of the observed indicator variables (often relying on the approach by Vale and Maurelli, 1983), they omit an important aspect: The genuine factor analytic structure of SEM defines the indicator variables as the sum of the weighted latent factors and error terms ***X*** = **Λ*ξ*** + ***ε***, with ***X*** containing the indicator variables, **Λ** collecting the loadings of the latent factors ***ξ*** and ***ε*** containing the error terms. Correspondingly, non-normality in the indicator variables can originate from non-normally distributed latent factors or from non-normally distributed errors (Auerswald & Moshagen, 2015). This distinction between non-normal latent factors and errors has been addressed by asymptotic robustness theory (Amemiya & Anderson, 1990; Browne, 1987; Browne & Shapiro, 1988; Mooijaart & Bentler, 1991; Shapiro, 1987), which specifies conditions under which normal theory test statistics asymptotically follow a chi-square distribution if the sample size *N* → ∞, even if the normality assumption is violated. For example, Amemiya and Anderson (1990) considered the following model:

1 $${\boldsymbol{x}}_i=\boldsymbol{\mu} +\boldsymbol{\Lambda} {\boldsymbol{f}}_i+{\boldsymbol{u}}_i.$$

For 1 ≤ *i* ≤ *N*, ***x***_i_ is the observable *p* × 1 random vector, **μ** is a *p* × 1 parameter vector, **Λ** is a *p* × *h* loading matrix, ***f***_*i*_ is an *h* × 1 unobservable factor vector, and ***u***_*i*_ is a *p* × 1 unobservable error vector. In this case, normal theory test statistics are asymptotically chi-square distributed if the ***f***_*i*_ are independently and identically distributed (i.i.d.) with any distribution with finite variance, if the ***u***_*i*_ are i.i.d. with any distribution with finite variance, and the *p* components of ***u***_*i*_ are independent.

However, it is important to note that asymptotic robustness theory only guarantees that the test statistics follow a chi-square distribution asymptotically, so that the actual distribution in finite (and realistic) samples might diverge substantially. Whereas few simulation studies have investigated the effect of the underlying multivariate distribution on the manifest variables by generating data based on non-normal latent factors (e.g., Molenaar et al., 2010; Schmitt et al., 2006), these did not systematically compare the effect of non-normal latent factors versus non-normal errors. An exception is a small simulation study by Auerswald and Moshagen (2015), where data were generated based on non-normally distributed latent factors as well as on non-normally distributed errors, respectively. This study provided evidence that the source of non-normality has an important effect on the uncorrected as well as on the Satorra–Bentler scaled test statistic in finite samples. Specifically, they found that the type I error rates of these statistics are inflated in the case of non-normal latent variables but not in the case of non-normal errors. However, these sources of non-normality were commonly confounded in previous simulation studies. To gain a more profound understanding of how the multivariate distribution (i.e., the distribution of latent factors and errors) affects corrections to the test statistics in finite samples, we thus extended the study of Auerswald and Moshagen (2015), which was limited by investigating the behavior for one sample size (*N* = 500) and by considering only one degree of non-normality. The present study relies on more comprehensive analyses including several test statistics correcting not only the mean but higher-order moments (see below for details) and investigates the effects of different extents of non-normality in sample sizes that are commonly encountered in substantive research.

The present study thus aims to answer the question to which extent the results from previous robustness studies are valid if the source of non-normality is considered. To this end, we relied on the NOTAMO (NOrmal To Arbitrary MOments) algorithm (Auerswald, 2017), which can be used to generate marginal distributions (i.e., the distribution of the indicator variables) sharing prespecified central moments that nevertheless differ in their multivariate distributions. NOTAMO induces non-normality in latent factors or errors, so the source of non-normality can be manipulated. We investigated the effect of the source of non-normality on the uncorrected LRT statistic based on normal theory ML estimation as well as on several corrections adjusting different central moments. This is of particular interest given that NOTAMO allows one to manipulate the source of non-normality while holding the central moments of the marginal distributions constant. Beyond considering moment-corrected test statistics, we also investigated an approach that directly estimates the underlying limiting chi-square mixture distribution to draw inferences.

The test statistic to evaluate the overall model fit in SEM depends on the sample estimate of the minimum of the fit function $\hat{F}={F}\left(\boldsymbol{S},\boldsymbol{\Sigma} \left(\hat{\boldsymbol{\theta}}\right)\right)$, where the parameter estimates $\hat{\boldsymbol{\theta}}$ are determined in such a way that they minimize the discrepancy between the model-implied variance-covariance matrix $\boldsymbol{\Sigma} \left(\hat{\boldsymbol{\theta}}\right)$ and the empirical variance-covariance matrix ${\boldsymbol{S}}$ (for details, see Bollen, 1989). ML estimates can be obtained based on the weighted least squares (WLS) fit function

2 $${\hat{F}}_{WLS}=\left(\boldsymbol{s}-\boldsymbol{\sigma} \left(\hat{\boldsymbol{\theta}}\ \right)\right)^{\prime }{\boldsymbol{W}}^{-1}\left(\boldsymbol{s}-\boldsymbol{\sigma} \left(\hat{\boldsymbol{\theta}}\right)\right),$$

where ***s*** and $\boldsymbol{\sigma} \left(\hat{\boldsymbol{\theta}}\right)$ represent a vector with the unique elements of ***S*** and $\boldsymbol{\Sigma} \left(\hat{\boldsymbol{\theta}}\right)$, respectively, and ***W*** denotes a weight matrix. When the unique elements of $\boldsymbol{\Sigma} \left(\hat{\boldsymbol{\theta}}\right)$ are used as weights, the WLS estimates are asymptotically equivalent to the estimates obtained based on ML estimation given by

3 $${\hat{F}}_{ML}=\ln |\ \boldsymbol{\Sigma} (\hat{\boldsymbol{\theta}})|-\ln |\mathbf{S}|+ tr[\mathbf{S}\ \boldsymbol{\Sigma} {(\hat{\boldsymbol{\theta}})}^{-\mathbf{1}}]-p$$

with *p* indicating the number of manifest variables (for details see Bollen, 1989; Browne, 1974). That means that although the fit functions in Eqs. 2 and 3 differ, both can be used to obtain ML estimates. Note that both fit functions refer to models without mean structure (see e.g., Hayashi et al., 2007 for details regarding fit functions for mean and covariance structures).

Given the validity of a set of assumptions, the asymptotic distribution of the (Wishart) LRT statistic ${T}_{ML}={\hat{F}}_{ML}\left(N-1\right)$ under the null hypothesis (i.e., the population variance-covariance matrix equals the model-implied variance-covariance matrix) follows a chi-square distribution with *df* = *p*^∗^ − *q* degrees of freedom with ${p}^{\ast }=\frac{p\left(p+1\right)}{2}$ and *q* as the number of free model parameters (for details, see Bollen, 1989). If the asymptotic robustness condition holds, *T*_*ML*_ also follows the same chi-square distribution as *N* → ∞. More generally, the test statistic can be shown to follow a weighted mixture distribution of independent chi-square variables with 1 degree of freedom each

4 $${T}_{ML}\overset{L}{\to}\sum_{j=1}^{df}{w}_j{\chi}^2(1)$$

where *w*_*j*_ denotes the weights (Satorra & Bentler, 1994). The weights are the non-null eigenvalues of **UΓ**, where **U** is the residual weight matrix defined as

5 $$\boldsymbol{U}={\boldsymbol{W}}^{-1}-{\boldsymbol{W}}^{-1}\mathbf{\Delta }{\left(\mathbf{\Delta }^{\prime} {\boldsymbol{W}}^{-1}\mathbf{\Delta }\right)}^{-1}\mathbf{\Delta }^{\prime }{\boldsymbol{W}}^{-1}$$

with $\boldsymbol{\Delta }=\boldsymbol{\Delta }\left(\theta \right)=\frac{\partial \sigma }{\partial {\theta}^{\prime }}$ denoting the *p*^∗^ × *q* Jacobian matrix and **Γ** referring to the asymptotic variance-covariance matrix of the distribution of $\sqrt{\left(N-1\right)}\left(\boldsymbol{s}-{\boldsymbol{\sigma}}_{\mathbf{0}}\right)$, where ***σ***_**0**_ is a vector with the unique elements of the population variance-covariance matrix, **Σ**_**0**_ (Browne, 1984; Satorra & Bentler, 1994).

As is immediately evident from Eq. 4, the actual distribution of *T*_*ML*_ can only be appropriately described by an unweighted chi-square distribution when all weights are equal to one. If the weights disperse around one, as happens, for instance, when the normality assumption is violated (Brosseau-Liard et al., 2012; Satorra & Bentler, 1994), the test statistic follows a chi-square weighted mixture distribution. In such cases, drawing inferences from an unweighted reference chi-square distribution leads to incorrect conclusions.

Based on this observation, the core idea of many corrected test statistics is to rely on the (unweighted) chi-square reference distribution to draw inferences but to adjust *T*_*ML*_ by the estimated weights, so that certain moments of the distribution are asymptotically equal to the respective moments of the unweighted reference chi-square distribution. The Satorra–Bentler scaled chi-square test statistic *T*_*M*_ adjusts the mean of the test statistic leading to an approximate chi-square distribution with asymptotically correct mean (i.e., the degrees of freedom of the test statistic):

6 $${T}_M=\frac{T_{ML}}{c}$$

with the scaling factor $c=\frac{tr\left(\hat{\boldsymbol{U}}\hat{\boldsymbol{\varGamma}}\right)}{df}$ and $tr\left(\hat{\boldsymbol{U}}\hat{\boldsymbol{\varGamma}}\right)$ as the expected value of the asymptotic distribution of the test statistic (Satorra & Bentler, 1994). As issues regarding the performance of *T*_*M*_ in small samples have been reported in the literature (e.g., Nevitt & Hancock, 2004; Savalei, 2010), we applied the Bartlett (1950) correction to *T*_*M*_ leading to *T*_*MB*_ given by

7 $${T}_{MB}={T}_M\left(1-\left[\frac{2p+4h+5}{6\left(N-1\right)}\right]\right),$$

where *h* represents the number of latent factors.

Rather than just adjusting the mean, the Satorra–Bentler adjusted chi-square test statistic *T*_*MV*1_ given by

8 $${T}_{MV1}=\frac{d}{tr\left(\hat{\boldsymbol{U}}\hat{\boldsymbol{\Gamma}}\right)}{T}_{ML}$$

results in an approximate chi-square distribution of the test statistic with $d=\frac{{\left[ tr\left(\hat{\boldsymbol{U}}\hat{\boldsymbol{\Gamma}}\right)\right]}^2}{tr\left[{\left(\hat{\boldsymbol{U}}\hat{\boldsymbol{\Gamma}}\right)}^2\right]}$ degrees of freedom and asymptotically correct mean and variance (Satorra & Bentler, 1994).

A related correction scales and shifts the underlying distribution (Asparouhov & Muthén, 2010). This correction leads to a test statistic with *df* degrees of freedom and asymptotically correct mean (i.e., *df*) and variance (i.e., 2 *df*). The corrected test statistic is defined as

9 $${T}_{MV2}={T}_{ML}\sqrt{\frac{df}{tr\left[{\left(\hat{\boldsymbol{U}}\hat{\boldsymbol{\Gamma}}\right)}^2\right]}}+ df-\sqrt{\frac{df{\left[ tr\left(\hat{\boldsymbol{U}}\hat{\boldsymbol{\Gamma}}\right)\right]}^2}{tr\left[{\left(\hat{\boldsymbol{U}}\hat{\boldsymbol{\Gamma}}\right)}^2\right]}}.$$

Beyond correcting the mean and variance, the third moment adjusted test statistic *T*_*MS*_ (Lin & Bentler, 2012) adjusts the mean and the skewness of the test statistic via

10 $${T}_{MS}=\frac{v}{tr\left(\hat{\boldsymbol{U}}\hat{\boldsymbol{\Gamma}}\right)}{T}_{ML},$$

where $v=\frac{{tr\left[{\left(\hat{\boldsymbol{U}}\hat{\boldsymbol{\Gamma}}\right)}^2\right]}^3}{tr{\left[{\left(\hat{\boldsymbol{U}}\hat{\boldsymbol{\Gamma}}\right)}^3\right]}^2}$. The corrected test statistic *T*_*MS*_ asymptotically follows a chi-square distribution with *v* degrees of freedom and shares its mean and skewness values with the unweighted reference chi-square distribution.

Instead of correcting *T*_*ML*_ by adapting particular standardized moments so that it more closely follows the expected unweighted chi-square reference distribution, an alternative procedure is to rely on the uncorrected *T*_*ML*_, but to draw inferences from the proper asymptotic weighted mixture distribution as defined in Eq. 4. The weighted chi-square mixture distribution can be estimated using the (non-null) eigenvalues of $\hat{\boldsymbol{U}}\hat{\boldsymbol{\Gamma}}$ as weights *w*_*j*_. The resulting weighted mixture distribution has an expected value of $tr\left(\hat{\boldsymbol{U}}\hat{\boldsymbol{\Gamma}}\right)$ and a variance of $tr\left[{\left(\hat{\boldsymbol{U}}\hat{\boldsymbol{\Gamma}}\right)}^2\right]$ (for details, see Satorra & Bentler, 1994). However, by constructing the mixture distribution using all weights, the resulting distribution should approximate the actual distribution of *T*_*ML*_ concerning all higher order moments, rather than just the mean, variance, and/or skewness. Throughout this paper, we refer to this test statistic as *T*_*mix*_. Note that *T*_*mix*_ has the same chi-square value as *T*_*ML*_; however, the *p*-value of the former might differ from that of the latter because of the different reference distributions involved.

Finally, we also considered derived fit indices, i.e., the root mean square error of approximation (RMSEA; Steiger, 2016; Steiger & Lind, 1980) and the comparative fit index (CFI; Bentler, 1990). To maintain comparability with previous robustness studies, we additionally simulated control conditions directly manipulating the distribution of the indicator variables by means of the Vale–Maurelli (VM; Vale & Maurelli, 1983) approach for non-normal data and by means of eigendecomposition for multivariate normal data.

Based on previous findings, we expected the uncorrected test statistic to perform best under conditions of multivariate normality and to observe inflated type I error rates with an increasing degree of non-normality, in particular when non-normality arises from non-normal latent variables. Whereas the corrections under scrutiny are expected to recover the true population value more closely regardless of the degree of non-normality (e.g., Curran et al., 1996; Tong & Bentler, 2013), we also expect an effect of the source of non-normality on these outcomes as indicated by Auerswald and Moshagen (2015).

## Methods

We created various experimental conditions to assess the effect of the source of non-normality on different test statistics by considering three non-normality conditions (*latent*, where non-normality in indicator variables originated from non-normal latent factors; *error*, where non-normality in indicator variables originated from non-normal errors; and *marginal*, where non-normality was directly induced in the indicator variables), four sample sizes (*N* = 200, 400, 600, and 1000), six test statistics (one uncorrected, four with corrected moments, and one estimating the limiting weighted mixture distribution), three degrees of kurtosis (*k* = 3, 10, and 17), and two specification statuses of the model (correct versus incorrect). We considered different measures of model fit, namely the rejection rates of the LRT statistic as well as the RMSEA and the CFI, as both depend on the LRT statistic and are thus affected by the analyzed corrections. Data generation and analysis were performed with the open-source software R (R Core Team, 2020) using the package *lavaan* (version: 0.6-6; Rosseel, 2012) for model estimation and the package *distrEx* (version 2.8.0; Ruckdeschel et al., 2019) to estimate the weighted mixture distribution.

### Population and analysis models

We defined a factor analytic model in the population with three latent factors and *p* = 15 indicator variables. Data generation was based on the variance-covariance matrix in the population **Σ**_**0**_ given by **Σ**_**0**_ = **ΛΦΛ**^′^ + **Θ**, where **Λ**′ represents the transposed matrix of loadings and **Φ** is the variance-covariance matrix of the latent factors. The elements of the diagonal residual matrix **Θ** were defined such that the respective squared loadings of **Λ** summed up with the respective residual term to one. We defined a correlated three-factor model with six nonzero secondary loadings

$$\boldsymbol{\Lambda} '=\left(\begin{array}{ccccccccccccccc}.7& .7& .5& .45& .40& 0& .25& 0& 0& 0& 0& 0& -.25& 0& 0\\ {}0& -.25& 0& 0& 0& .80& .65& .55& .50& .40& 0& 0& 0& .25& 0\\ {}0& 0& .25& 0& 0& 0& 0& 0& -.25& 0& .70& .60& .55& .50& .45\end{array}\right)$$

and a variance-covariance matrix between the factors of

$$\boldsymbol{\Phi} =\left(\begin{array}{ccc}1& & \\ {}.3& 1& \\ {}.2& .3& 1\end{array}\right).$$

In conditions involving correctly specified models, all secondary loadings were freely estimated, whereas a confirmatory factor analysis model with three factors and no secondary loadings was estimated in conditions considering misspecified models. The conditions involving misspecifications were associated with a population minimum of the fit function of *F*_0_ = 0.328. Table 1 further shows the expected power (Jobst et al., in press; Moshagen & Erdfelder, 2016) of the LRT statistic as well as the population values of descriptive indices of model fit.

**Table 1:** Misspecification in the population, expected power, and population values of fit indices under misspecification

	Expected power				Fit indices in the population under misspecification	
Misspecification in the population	*N* = 200	*N* = 400	*N* = 600	*N* = 1000	*RMSEA*_*0*_	*CFI*_*0*_
*F*_*0*_ = 0.328	0.986	> 0.999	> 0.999	> 0.999	0.061	0.884

### Data generation

Based on the population model described above, we drew 1000 random samples each (only valid solutions that converged were considered) with *N* = 200, 400, 600, and 1000 observations, respectively, mimicking common sample sizes in psychological research using factor analytic methods (e.g., DiStefano & Hess, 2005; Jackson et al., 2009).

All generated observed variables *X*_*i*_ with 1 ≤ *i* ≤ *p* had a kurtosis of either *k* = 3, 10, or 17 representing values that were observed in substantive research (e.g., Blanca et al., 2013; Cain et al., 2017). We specified the distribution of the indicator variables by either manipulating the multivariate distribution—based on non-normal errors or non-normal latent factors—or by directly drawing samples from marginal distributions with the respective kurtosis. In conditions with *k* = 10 and *k* = 17, respectively, the VM approach was used to induce non-normality in the marginal distributions. Moreover, we generated a multivariate normal control condition based on eigendecomposition (*marginal* condition with *k* = 3). Note that a marginal kurtosis of three and a skewness of zero can arise when data are multivariate normal, which we realized in the marginal condition for *k* = 3. Nevertheless, it is also possible to obtain multivariate non-normal data exhibiting the same values regarding skewness and kurtosis as a multivariate normal distribution (i.e., skewness of zero and kurtosis of three) but differing in higher order moments, which we realized in conditions with *k* = 3 under latent and error non-normality. This setup thus allows for the comparison between both multivariate normal and multivariate non-normal data sharing their skewness and kurtosis values.

Non-normality based on the multivariate distribution was created relying on the NOTAMO framework (Auerswald, 2017). Within this framework, the indicator variables *X*_*i*_ are defined as the sum of two random variables *L*_*i*_ and *E*_*i*_. All *L*_*i*_ are correlated amongst each other, whereas all *E*_*i*_ are independent from all other *E*_*i*_ as well as from all *L*_*i*_*.* Depending on the particular non-normality condition, the distributions of *L*_*i*_ and *E*_*i*_ vary: In conditions with non-normal latent variables, all *L*_*i*_ follow a non-normal distribution and all *E*_*i*_ are standard normally distributed. In conditions with non-normal errors, all *L*_*i*_ are standard normally distributed, but all *E*_*i*_ follow a non-normal distribution. Non-normal *L*_*i*_ and *E*_*i*_, respectively, were generated with the NORTA algorithm (Cario & Nelson, 1997) requiring an inverse cumulative distribution function *F*^−1^ as input. We used NOTAMO to identify a suitable inverse cumulative distribution function for each random variable *X*_*i*_ that complied with the prespecified central moments. NOTAMO defines the target inverse cumulative distribution function *F*^−1^ as a weighted sum described by the following quantile mixture distribution:

11 $${F}^{-1}=\sum_{m=1}^l{\beta}_m{F}_m^{-1},$$

with *β*_1_, …, *β*_*l*_ as positive weights and $\sum_{m=1}^l{\beta}_m=1$*.* Depending on the experimental condition, we varied the input inverse cumulative distribution functions ${F}_m^{-1}$ across conditions. In conditions with *k* = 3 and non-normal latent factors, we used a t-distribution with 4.1 degrees of freedom and a uniform distribution on the interval [0, 1]. For *k* = 3 and non-normal errors, a cubic standard normal distribution, a uniform distribution on the interval [0, 1], and a standard normal distribution were used to define *F*^−1^. In conditions with *k* = 10 and non-normal errors as well as non-normal latent factors, we used a log-normal distribution with a mean of zero and standard deviation of one, as well as an exponential distribution with a rate of one, as input functions. The same two inverse cumulative distribution functions were used in conditions with *k* = 17 and non-normal latent factors. In conditions with non-normality in errors and *k* = 17, the input functions for the quantile mixture were a standard normal distribution and a mixture distribution based on a log-normal distribution and a negative log-normal distribution.

### Study outcomes

We used the percentage of empirical *p*-values equal or smaller than the nominal significance level of .05 (i.e., the empirical rejection rates) of the LRT statistic as an indicator of type I error when estimating correctly specified models and as an indicator of the actually achieved power when estimating misspecified models. Moreover, we evaluated the performance of the RMSEA and the CFI, because both fit indices directly depend on the LRT statistic. For both fit indices, we used the median across the 1000 replications as the respective point estimate. The RMSEA in the population is defined as

12 $$RMSE{A}_0=\sqrt{\frac{F_0}{df}}$$

with *F*_*0*_ indicating the population discrepancy and thus setting the minimum of the fit function in relation to the degrees of freedom of the model (Steiger, 2016; Steiger & Lind, 1980). The CFI (Bentler, 1990) expresses the proportional reduction in misfit by comparing the minimum of the fit function based on a null model (*nm*)—where all covariances equal zero—against the minimized fit function of the hypothesized model leading to

13 $$CF{I}_0=\frac{F_{nm}-{F}_0}{F_{nm}}.$$

We obtained sample estimates for the uncorrected test statistic *T*_*ML*_ based on

14 $$RMSEA=\sqrt{\max \left(0,\frac{T_{ML}- df}{\left(N-1\right) df}\right)}$$

and

15 $$CFI=1-\frac{T_{ML}- df}{T_{nm}-d{f}_{nm}}.$$

The RMSEA and CFI of *T*_*mix*_ were also computed based on Eqs. 14 and 15, respectively, but *df* was replaced by $tr\left(\hat{\boldsymbol{U}}\hat{\boldsymbol{\Gamma}}\right)$. Table 2 provides the sample formulas of both fit indices considering the moment-based corrections to *T*_*ML*_. The uncorrected as well as the corrected sample estimates approximate the population values as in Eqs. 12 and 13, respectively (for details, see Brosseau-Liard et al., 2012; Brosseau-Liard & Savalei, 2014; Savalei, 2018). Within any one condition, the underlying correction approach was also applied to the null model.

**Table 2:** Sample formulas of fit indices regarding corrections to *T*_*ML*_

Test statistic	RMSEA sample formula	CFI sample formula
*T*_*MB*_	$\sqrt{\max\ \Big(0,\frac{\mathrm{c}\left({T}_{MB}- df\right)}{\left(N-1\right) df}}\Big)$	$1-\max \left(0,\frac{c\left({T}_{MB}- df\right)}{c_{nm}\left({T}_{M{B}_{nm}}-{df}_{nm}\right)}\right)$
*T*_*MV1*_	$\sqrt{\max\ \left(0,\frac{b\left({T}_{MV1}-d\right)}{\left(N-1\right)d}\right)}$ with $b=\frac{{tr}\left(\hat{U}\hat{\Gamma}\right)}{d}$	$1-\max \left(0,\frac{b\left({T}_{MV1}-d\right)}{b_{nm}\left({T}_{MV{1}_{nm}}-{d}_{nm}\right)}\right)$
*T*_*MV2*_	$\sqrt{\max\ \left(0,\frac{a\left({T}_{MV2}- df\right)}{\left(N-1\right) df}\right)}$ with *a* =$\sqrt{\frac{tr\left[{\left(\hat{U}\hat{\Gamma}\right)}^2\right]}{df}}$	$1-\max \left(0,\frac{a\left({T}_{MV2}- df\right)}{a_{nm}\left({T}_{MV{2}_{nm}}-{df}_{nm}\right)}\right)$
*T*_*MS*_	$\sqrt{\max\ \left(0,\frac{g\left({T}_{MS}-v\right)}{\left(N-1\right)v}\right)}$ with $g=\frac{{tr}\left(\hat{U}\hat{\Gamma}\right)}{v}$	$1-\max \left(0,\frac{g\left({T}_{MS}-v\right)}{g_{nm}\left({T}_{M{S}_{nm}}-{v}_{M{S}_{nm}}\right)}\right)$

## Results

To maintain scope and increase clarity, we only present an illustrative subset of the results. The complete data and further results are available as supplementary materials in the open science framework (OSF) repository at https://osf.io/fxnsu/.

### Effect on empirical rejection rates and empirical power

**Fig. 1:**
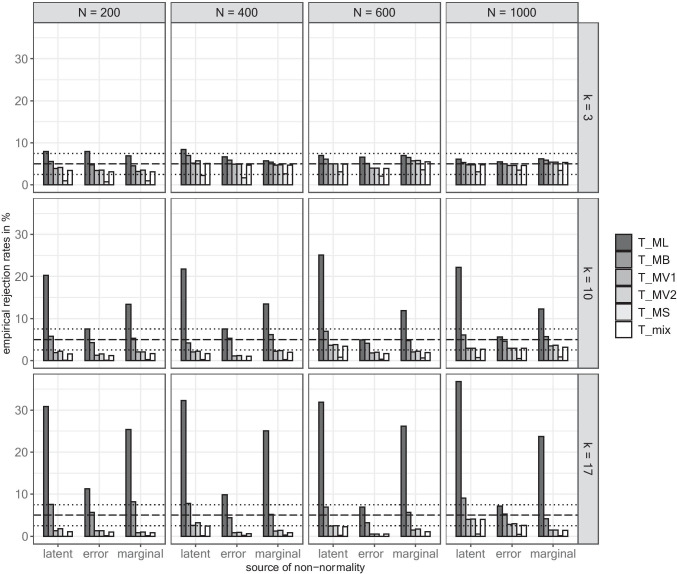
Empirical rejection rates. *Note.* The dashed line illustrates the nominal significance level of 5.0% and the dotted lines illustrate the robustness criterion of 2.5% and 7.5%.

We relied on the liberal robustness criterion suggested by Bradley (1978) deeming rejection rates within the interval [*α* ± 0.5*α*] acceptable (i.e., [2.5%, 7.5%] based on a significance level α of .05). The underlying multivariate distribution revealed no relevant effect in conditions with *k* = 3. The rejection rates of most test statistics were close to the nominal level of 5.0% (see Fig. 1). Exceptions pertained to *T*_*ML*_ and *T*_*MS*_ in small samples, where the rejection rates were above the robustness criterion for *T*_*ML*_ and below the robustness criterion for *T*_*MS*_.

With an increasing extent of non-normality (i.e., *k* > 3), *T*_*ML*_ showed increasing empirical rejection rates regardless of sample size—in particular in conditions with latent non-normality—with empirical rejection rates up to 36.8%. By contrast, the rejection rates were much lower under error non-normality and were above the robustness criterion only when *k* = 17, but decreased with increasing sample size. *T*_*MB*_ yielded adequate rejection rates and exhibited only a slight tendency to overreject a fitting model when *k* = 17 in the case of latent non-normality for *N* = 1000 and under marginal non-normality for *N* = 200. There were only minor differences between the remaining test statistics, which tended to underreject models in all conditions when *k* > 3. Whereas *T*_*MS*_ indicated a too optimistic fit across conditions, all remaining corrected test statistics showed acceptable rejection rates with increasing sample size if only a medium extent of non-normality was present. However, they exhibited rejection rates below the robustness criterion in conditions with *k* = 17, especially under error and marginal non-normality.

Based on the misspecification in the population, the expected power to reject the models was at least 0.986 (see Table 1). As summarized in Table 3, *T*_*ML*_ and *T*_*MB*_ closely recovered the expected power across all conditions. When *N* = 200, *T*_*MS*_ was associated with very low power regardless of the extent of kurtosis. The remaining test statistics yielded a power close to the expected values in small samples when *k* = 3, but too few rejections occurred with an increasing extent of non-normality, in particular under latent and marginal non-normality. As the sample size increased, the empirical power was generally adequate. Thus, the results concerning power generally mirror the results concerning the empirical rejection rates of correctly specified models by suggesting that all approaches other than *T*_*ML*_ and *T*_*MB*_ show a tendency to retain an incorrect model as non-normality increases.

**Table 3:** Empirical power in %

Test statistic	*k*	*N* = 200			*N* = 400		
		Source of non-normality			Source of non-normality		
		Latent	Error	Marginal	Latent	Error	Marginal
*T*_*ML*_	3	98.7	99.1	98.5	100.0	100.0	100.0
	10	98.9	99.0	98.9	100.0	100.0	100.0
	17	99.0	99.8	99.5	100.0	100.0	100.0
*T*_*MB*_	3	98.2	98.4	98.0	100.0	100.0	100.0
	10	95.9	98.7	98.1	100.0	100.0	100.0
	17	95.6	99.7	98.3	100.0	100.0	100.0
*T*_*MV1*_	3	96.9	97.5	97.6	100.0	100.0	100.0
	10	87.5	94.0	91.7	99.9	99.9	100.0
	17	81.1	95.3	83.4	99.4	99.4	99.7
*T*_*MV2*_	3	96.9	97.5	97.6	100.0	100.0	100.0
	10	89.3	94.6	93.1	99.9	99.9	100.0
	17	83.4	95.9	86.3	100.0	99.5	99.9
*T*_*MS*_	3	86.9	90.7	92.7	99.4	100.0	100.0
	10	37.4	65.5	54.7	90.0	95.9	97.0
	17	26.8	63.8	28.8	78.9	90.5	87.3
*T*_*mix*_	3	96.7	97.2	97.3	100.0	100.0	100.0
	10	85.2	93.4	91.2	99.9	99.9	100.0
	17	79.0	94.7	81.0	99.4	99.4	99.6

To avoid redundancies, empirical power for larger sample sizes was not displayed as the values were close to 100% across conditions—except for *T*_*MS*_ in conditions with *N* = 600 and *k* = 17 under latent non-normality, where the observed power was 90.9%.

### Effect on RMSEA

To summarize (see supplement for details), no effect of the multivariate distribution occurred for *k* = 3, whereas in conditions with *k* > 3 the point estimates of all test statistics were larger under marginal and latent non-normality compared to error non-normality. In general, the approximation of the population RMSEA_0_ improved in larger samples across test statistics and sources of non-normality.

**Fig. 2:**
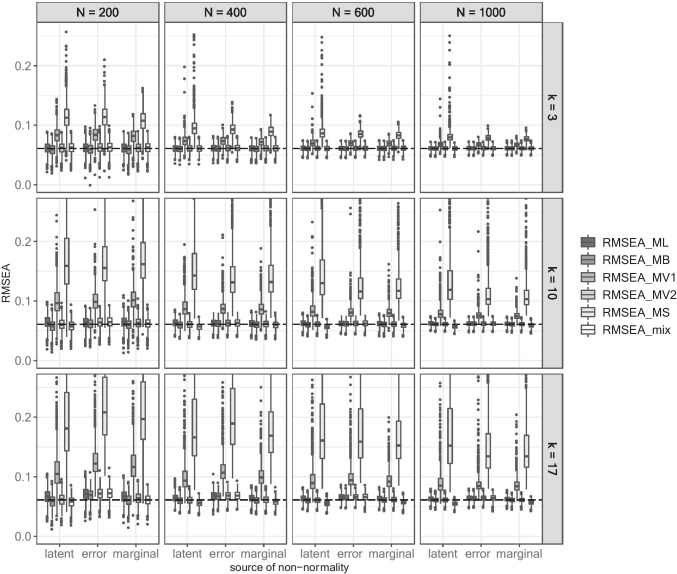
RMSEA in misspecified models per source of non-normality. *Note.* Each boxplot includes the values observed in 1000 replications. Values larger than 0.260 are not displayed, leading to 2762 non-displayed values (53 values for RMSEA_MV1_ and 2709 for RMSEA_MS_). The dashed line illustrates the population RMSEA_0_.

In misspecified models (Fig. 2), the point estimates of RMSEA_ML_, RMSEA_MB_, RMSEA_MV2_, and RMSEA_mix_ closely recovered RMSEA_0_ with a maximum difference between 0.008 and 0.011 depending on the involved test statistic. In contrast, the maximum difference was 0.06 for RMSEA_MV1_ and 0.147 for RMSEA_MS_. Again, no effect of the source of non-normality was evident in conditions with *k* = 3. However, RMSEA_MV1_ and RMSEA_MS_ distinctly differed from the population value and although this difference diminished with increasing sample size, the point estimates still exhibited a positive bias even in the largest sample size condition. When *k* = 10, the effect of the source of non-normality was rather small, yet a more pronounced pattern could be observed in conditions with *k* = 17: Whereas RMSEA_ML_ was virtually unaffected by the source of non-normality, the remaining test statistics yielded larger values in error non-normality conditions compared to both other sources of non-normality.

### Effects on CFI

Similar to empirical rejection rates and the RMSEA, no effect of the source of non-normality on the CFI in correctly specified models occurred in conditions with *k* = 3 (see supplement for details). In conditions with larger kurtosis, all test statistics exhibited smaller point estimates under latent and marginal non-normality than under error non-normality, especially in small samples.

**Fig. 3:**
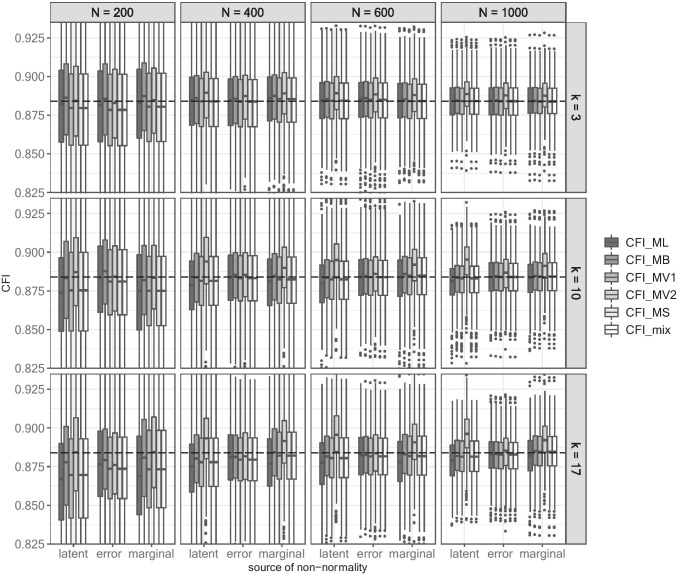
CFI in misspecified models per source of non-normality. *Note*. Each boxplot includes the values observed in 1000 replications. The dashed line illustrates the population CFI_0_. Values smaller than 0.830 and larger than 0.930 are not displayed, leading to 9732 non-displayed values (1708 values for CFI_ML_, 1642 values for CFI_MB_, 1489 values for CFI_MV2_, and 1631 non-displayed values each for CFI_MV1_, CFI_MS_, and CFI_mix_).

In misspecified models (see Fig. 3), the maximum difference between the CFI point estimate and CFI_0_ was −0.017 for CFI_ML_, −0.006 for CFI_MB_, 0.012 for CFI_MV2_, and −0.014 for CFI_MV1_, CFI_MS_, and CFI_mix_, respectively. The effect of the source of non-normality became visible in small samples when *k* > 3: CFI_ML_, CFI_MV1_, CFI_MS_, CFI_mix_ provided a closer approximation of CFI_0_ under error non-normality than under latent non-normality. However, the observed bias diminished with increasing sample size. In contrast, CFI_MB_ was virtually unaffected by the source of non-normality across conditions. A different pattern occurred for CFI_MV2_, where larger values were observed under marginal and latent non-normality than under error non-normality. As the point estimates increased with increasing sample size, this led in turn to a close approximation of CFI_0_ under marginal and latent non-normality in small samples and to virtually unbiased point estimates under error non-normality in larger samples.

## Discussion

Non-normal data regularly occur in substantive research, so yielding valid test statistics and descriptive indices of model fit under such conditions is of particular importance. Whereas a number of corrections to the LRT statistic (and hence, derived fit indices) has been proposed, previous robustness studies usually created non-normality by manipulating the marginal distributions only and thus did not consider the source of non-normality. The present study provides evidence that the uncorrected test statistic, four corrected test statistics, and one test statistic based on both the weighted chi-square mixture distribution and the derived fit indices are affected by the source of non-normality in finite samples, even when the manifest variables exhibited the same levels of kurtosis. Note that the manipulation of other standardized moments such as skewness would also induce non-normality in manifest variables. However, studies indicate that psychological variables exhibit a larger range of kurtosis values compared to skewness values. Additionally, these variables show a wider range of kurtosis values regarding leptokurtic distributions compared to platykurtic distributions (Blanca et al., 2013; Cain et al. 2017). Hence, we decided to investigate non-normality conditions based on leptokurtic data allowing for the generation of data sets distinctly differing in the extent of non-normality but still representing values that can be observed by substantive researchers (see e.g., Curran et al., 1996).

In line with previous robustness studies (e.g., Curran et al., 1996; Nevitt & Hancock, 2004), the uncorrected ML test statistic was associated with inflated type I error rates in the case of non-normally distributed data. However, when considering the source of non-normality, we showed that non-normal errors do not lead to increased rejection rates, which is consistent with the findings of Auerswald and Moshagen (2015). Thus, the uncorrected ML test statistic appears to be robust in finite samples when non-normality arises from non-normal errors but not when non-normality arises from non-normal latent variables.

All corrected test statistics were also affected by the source of non-normality, albeit to a smaller extent as compared to the uncorrected *T*_*ML*_. In particular, the Satorra–Bentler scaled test statistic with Bartlett correction (*T*_*MB*_) performed well across conditions by closely recovering the nominal significance level in correct models and closely approximating the expected power to reject incorrect models. By contrast, all remaining corrections under scrutiny showed rejection rates below the nominal significance level and a lower statistical power than expected. Marginal non-normality showed both of these effects, whereas the former was especially apparent in conditions of error non-normality and the latter occurred primarily under latent non-normality.

Correcting *T*_*ML*_ by the first standardized moment (i.e., mean) greatly improved its performance, whereas correcting further moments generally led to a tendency to retain models. This is unexpected as—from a theoretical point of view—corrections of higher-order moments should result in further improvements. A similar pattern of results was also evident for the approach to draw inferences from the estimated limiting mixture distribution. As this approach does not correct for particular standardized moments but directly estimates the underlying weighted mixture distribution, we expected a superior performance. The results, however, show that corrections of higher order moments, and especially the estimated weighted chi-square mixture distribution—where all moments should be correct, as the underlying distribution is directly estimated—generally were associated with an underestimation tendency, thus leading to an inadequate type I error control and a lack of statistical power. A possible explanation for the comparatively poor performance of all approaches attempting to correct for additional moments beyond the mean might lie in unreliabilities regarding the estimation of the weights via the eigenvalues of $\hat{\boldsymbol{U}}\hat{\boldsymbol{\Gamma}}$. The estimation errors of these weights have more severe consequences in non-linear corrections (such as the corrections for higher order moments) than in linear corrections (such as the correction for the first standardized moment), so the correction applied in *T*_*MB*_ might be less affected by incorrectly estimated weights, in turn leading to the observed superior performance.

Beyond the LRT statistic itself, we also investigated derived descriptive fit indices computed from the respective uncorrected or corrected test statistics. Concerning the RMSEA in correctly specified models, the source of non-normality had an effect on all versions of RMSEA but its magnitude varied across the analyzed test statistics. In misspecified models, all RMSEA based on corrected test statistics were affected by the source of non-normality by yielding larger values in error non-normality conditions than both other sources of non-normality; however, the observed bias was generally small to moderate. Exceptions pertain to RMSEA_MV1_ and RMSEA_MS,_ which strongly overestimated the population RMSEA_0_ leading to a too negative fit evaluation.

Concerning the CFI in correctly specified models, smaller point estimates occurred under marginal and latent non-normality compared to error non-normality, especially in small samples, regardless of the underlying test statistic. An effect of the source of non-normality also became evident in misspecified models, where CFI_ML_, CFI_MV1_, CFI_MS_, and CFI_mix_ showed a stronger bias under latent compared to error non-normality. Nevertheless, with increasing sample size the bias diminished for all test statistics except for CFI_MV2_, whose bias depended on the sample size and the source of non-normality.

## Conclusion

To assess model fit in substantive research, it is recommended to not rely on a single criterion but to consider various measures of fit (for an overview, see West et al., 2012). Whereas we showed that *T*_*ML*_ is virtually unbiased when non-normality arises from non-normal errors, the source of non-normality is unknown in practice. In case of non-normal data, we thus recommend relying on the Satorra–Bentler scaled (i.e., mean-corrected) test statistic with Bartlett correction (*T*_*MB*_), which performed satisfactorily throughout conditions regardless of the particular source of non-normality. Generally, all remaining corrections considered herein (*T*_*MV1*_, *T*_*MV2*_, *T*_*MS,*_ *T*_*mix*_) revealed systematic biases in at least some conditions, in particular when latent variables were non-normal. Thus, we recommend against their use. This general recommendation also extends when considering RMSEA or CFI as descriptive indices of fit, because indices based on *T*_*MV1*_, *T*_*MV2*_, *T*_*MS*_ did not perform well, whereas RMSEA and CFI based on *T*_*MB*_ performed satisfactorily overall. The results also indicate that a better approximation can be obtained when using the degrees of freedom as obtained by $tr\left(\hat{\boldsymbol{U}}\hat{\boldsymbol{\Gamma}}\right)$, as we have done for the *T*_*mix*_ approach. In general, we encourage researchers to consider distributional information such as the expected value and use unbiased sample estimates of descriptive fit indices.

To summarize, we demonstrated that the source of non-normality has an effect not only on the uncorrected but also on corrected test statistics, which is especially relevant as these corrections are used to deal with non-normal data. No general pattern could be identified because the particular effects on measures of fit depend on variables like the applied test statistic or the specification status of the model. However, the present work shows that some test statistics are rather robust regarding the source of non-normality, whereas others are strongly affected by non-normal latent factors but are not necessarily affected by non-normal errors. Although the six investigated test statistics showed varying patterns across the analyzed conditions, *T*_*MB*_ seems suitable to correct for non-normality regardless of the extent or source of non-normality and thus appears to be a reasonable choice to evaluate model fit in the presence of non-normal data. Concerning RMSEA and CFI as descriptive indices of fit, we suggest relying on robust versions based on *T*_*MB*_ approximating the same population value as versions of these indices based on the uncorrected ML LRT statistic.

## Declarations

### Open Practices Statement – Availability of data and materials

Data and supplemental materials of the current study are available in the Open Science Framework (OSF) at https://osf.io/fxnsu/. The simulation study was not preregistered.

### Open Practices Statement – Code availability

A script illustrating how the analyzed test statistics can be obtained is available in the Open Science Framework (OSF) at https://osf.io/fxnsu/. The code used to generate and analyze the data of the current study is available from the corresponding author on reasonable request.
